# Why the World Should Not Follow the Failed United States Model of Fighting Domestic Hunger

**DOI:** 10.3390/ijerph19020814

**Published:** 2022-01-12

**Authors:** Joel Berg, Angelica Gibson

**Affiliations:** Hunger Free America, New York, NY 10004, USA; agibson@hungerfreeamerica.org

**Keywords:** food insecurity, hunger, COVID-19, food banks

## Abstract

Many industrialized nations have followed the lead of the United States (US) in reducing workers’ wages and cutting government safety nets, while giving their populaces the false impression that non-governmental organizations can meet the food and basic survival needs of their low-income residents. The history of the last 50 years and the global COVID-19 pandemic demonstrate why that is a mistake, leading to vastly increased household food insecurity, poverty, and hunger. This paper takes a close look at US data to help to better understand the significant impact US federal government policy measures had on limiting hunger throughout the pandemic and how we can learn from these outcomes to finally end hunger in America and other developed nations. The top three policy prescriptions vital in ending household food insecurity in the US and industrialized countries are as follows: (1) to create jobs; raise wages; make high quality healthcare and prescription medicine free; and ensure that high quality childcare, education, transportation, and broad-band access are affordable to all; (2) to enact a comprehensive “Assets Empowerment Agenda” to help low-income people move from owing to owning in order to develop middle-class wealth; and (3) when the above two steps are inadequate, ensure a robust government safety net for struggling residents that provides cash, food, and housing assistance.

## 1. Introduction

Starting in the 1980s, residents of the United States (US) were told by the government and a compliant media [1] that the best way to address domestic hunger and poverty was through charity, one person at a time, one donated can of food at a time [2]. The increasing public focus on limited charity papered over government policies that promoted low wages such as by outsourcing jobs overseas and reduced safety net programs such as by cutting funding for food aid.

Over subsequent decades, even as evidence (such as rising rates of household food insecurity and poverty) mounted that nonprofit organizations (as non-government charities are generally called in the US) were inefficient and inadequate in addressing large, systematic problems such as food insecurity, industrialized democracies followed the US model by encouraging charitable responses by non-governmental organizations (NGOs)—such as food banks, food pantries, soup kitchens, food recue groups, mutual aid societies, and other non-governmental communal institutions—while reducing government protections and supports. The United States government defines households as food insecure, in the domestic context, when, some times during a year, “their access to adequate food is limited by a lack of money and other resources” [3].

This paper argues that such an approach is a tragic, evidence-adverse mistake and that both history and current events prove that major societal problems such as food insecurity can only be solved with extensive, coordinated, society-wide action, led by the only entity capable of organizing such action: each country’s national government.

## 2. History of Hunger in the United States

### 2.1. The Reduction of the Social Safety Net

Through US history, nonprofit and voluntary civic groups have played a larger role in the delivery of social services than in most nations, but, especially since US President Franklin Roosevelt’s New Deal in the 1930s, such non-governmental efforts were considered a supplement, not a replacement for, governmental programs. However, in the 1980s, US President Ronald Reagan began selling his populace the false notion that voluntary, under-funded, and largely uncoordinated private charity—as well as “trickle-down” economic activity (the theory that tax cuts to the wealthy would benefit the larger society)—could be a substitute for government and unilaterally make up for major failures in economic and social policy including high unemployment, declining wages, crushed unions, outsourced jobs, and slashed safety nets. Reagan and a coalition of Republicans and conservative Democrats in Congress cut food stamps (the main voucher program enabling low-income Americans to afford food) and the special supplemental nutrition program for women, infants, and children (WIC). They slashed funding for education, health, and housing. Altogether, the US Congressional Budget Office estimated that there were USD 110 billion reductions in social services between 1982 to 1986. A Reagan aide said private charities should “pick up the slack” caused by the cuts [4], and Reagan himself bragged that he gave 10 percent of his income to people in need, although questionable as to whether that was true [5]. 

**Figure 1:**
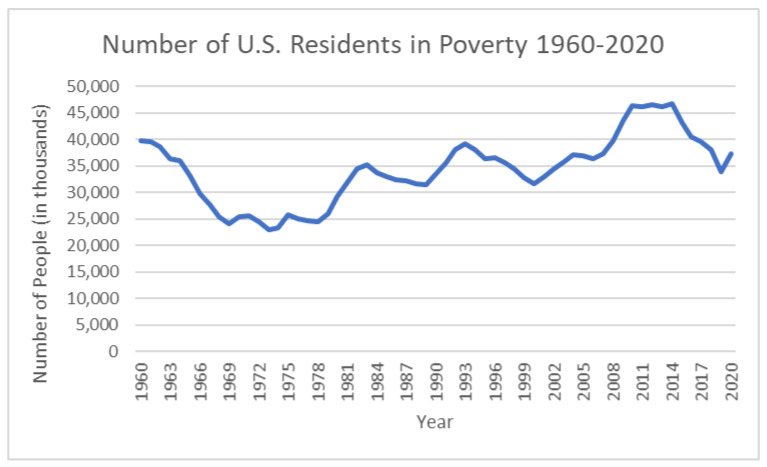
Number of US residents in poverty (in thousands), 1960–2020. Chart by Hunger Free America, based on publicly available US Census Bureau Data [7,8].

“Reaganomics”—as those policies were commonly referred to—failed in many respects. Poverty increased by 20 percent during Reagan’s first three years in office (Figure 1). The US government did not start measuring hunger (or, as the federal government calls it now, “low food security”) until 1995, but given the explosion in the number of feeding charities, it is clear from reports from social service providers that US hunger soared during that time [6]. Because Reagan dramatically increased military spending and gave massive tax cuts to the rich, the US budget deficit soared. Reagan then argued that such high deficits were one of the key reasons that the nation needed further cuts in social spending. 

As Figure 1 demonstrates, US poverty plummeted from 1960 to 1974, when broad-based economic growth was combined with a dramatic expansion of safety programs as part of the US War on Poverty but skyrocketed when the US middle class declined, and the social safety net was slashed.

### 2.2. An Increase in Charitable Feeding Programs

As a result, US society started to replace guaranteed access to government anti-poverty programs with private, under-funded, and largely uncoordinated charitable food banks, soup kitchens, and food pantries.

In 1980, there were only a few hundred charitable feeding programs across the US—most were soup kitchens and missions in the “skid rows” of large cities. According to Feeding America, the nation’s largest food bank network, there were over 58,000 private feeding programs across America in 2014 [9]. A majority of these are food pantries providing supplemental groceries to working families. Joel Berg has spent three decades visiting charities and speaking with people forced to go to them to obtain food and is repeatedly told that, since people who access these services often have to wait in long lines and deal with the internal struggles of embarrassment and humiliation about using these programs, services are often under-utilized, even by those with the most pressing economic needs. 

**Figure 2:**
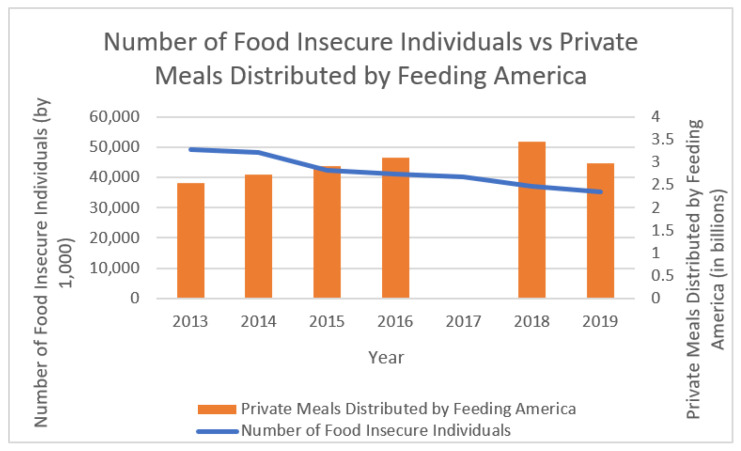
Number of food-insecure Americans compared to private meals distributed by Feeding America, 2013–2019. Charitable food data were calculated by Hunger Free America from statistics in the annual reports of the national NGO, Feeding America [11,12,13,14,15,16], and household food insecurity data are from the USDA’s 2020 Household Food Security report [3], both of which are publicly available. Feeding America data for 2017 is too limited to make an accurate calculation.

The dollar value of food provided by the charities is dwarfed by the amount of the reduced wages and anti-poverty programs cut. US charitable food distribution has continued to grow, but it has done little to solve the problem. The increase in food distribution by NGOs continued to grow in the US, encouraged by both Republican and Democratic Party leaders. There are numerous reasons why relying on food banks and other food charities to play a lead role in fighting hunger is insufficient. In her seminal 1999 book about the US charitable food system, *Sweet Charity, Emergency Food and the End of Entitlement,* sociologist Janet Poppendieck lists what she calls the “Seven Deadly ‘ins’” of the network: insufficiency (not enough food); inappropriateness (people do not get to pick what is best for their families); nutritional inadequacy (too much high-sugar, high-sodium, high-fat junk food); instability (feeding agencies cannot always predict when they will be open and when they will run out of food); inaccessibility (particularly in rural areas or for seniors, people with disabilities, and people without cars); inefficiency (the agencies require a massive, three-tier system just to give out free food); and indignity (at even the best-run agencies, it is usually degrading to obtain emergency food) [10]. Of all those problems, the insufficiency in the dollar amount of charitable food was dwarfed by the decrease in the food purchasing powers of low-income US residents due to wages reduction and social service cuts. As Figure 2 demonstrates, the monetary value of government-funded food dwarfs that of charitable food in the US.

**Figure 3:**
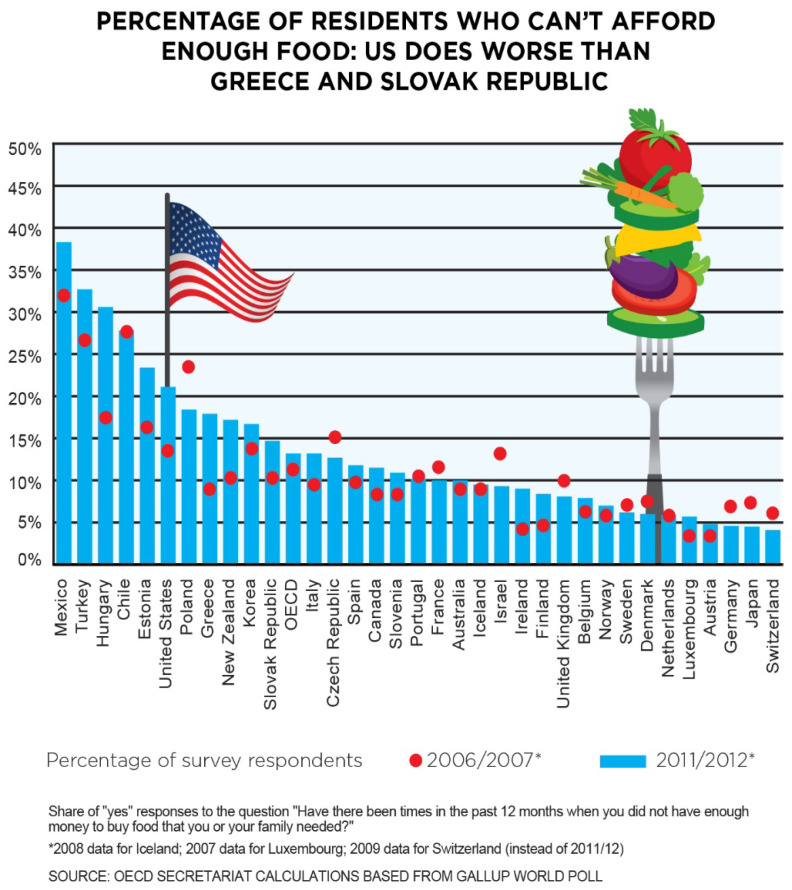
Comparison of food affordability in OECD countries. The percentage of the US population unable to afford an adequate supply of food was higher than most other OECD countries. Graph created by Hunger Free America using publicly available OECD data [17].

All told, the overall US response to hunger failed, as Figure 3 demonstrates. The percentage of the population unable to afford an adequate supply of food was greater in the US than Poland, Greece, South Korea, and the Slovak Republic.

While the level of overall, society-wide, food insecurity in any given nation is based largely on that nation’s level of socio-economic development, food insecurity at the household or individual level sometimes is a function of vast inequality of wealth and has very little to do with whether that nation is rich or poor overall. Vast wealth and vast poverty and hunger exist side by side in many countries, including the United States and India [18].

## 3. Charitable Food Bank Model’s Global Spread

Incorrectly believing that the charitable food bank model was a success, many industrialized nations started to emulate it.

Yet, as Graham Riches (2018) documents, starting in the 1980s and continuing today, many industrialized nations made the same mistakes as the US by increasing their societal reliance on food charities while reducing protections for workers and available social services. Riches, an Emeritus Professor of Social Work at the University of British Columbia, explains that in his home nation of Canada in the early 1980s, charitable breadlines were formed in response to their recession, high unemployment, and inadequate social insurance programs:

“Food banks began to pick up the pieces of a failing social safety net and became the early warning signs, symptoms, symbols of the retreat from the welfare state. The mantra of neo-liberalism hastened the withdrawal as deregulation, privatization, and the politics of the minimalist state gather pace… when the US food bank model of providing emergency food assistance was imported across the border to Canada, I was as shocked and curious then as I remain today” (p. 9) [19].

Riches then documents the expansion of food banks in other industrialized nations worldwide, led by the Global Food Bank Network, co-founded by Bob Forney, the former CEO of Feeding America, and former President of the Chicago Stock Exchange. Such efforts resulted in national food banks in 15 countries including in high and middle-income countries such as France, Australia, Brazil, the United Kingdom, Italy, India, South Korea, China, and Chile [20]. Operationally, many foreign food charities are directly modeled on their US counterparts, for example, co-author Berg visited a food bank in Peth, Australia, whose floor plan was literally supplied by a US food bank.

International food banks often share something else with their US counterparts: they give the misleading impression that they are fully solving the hunger problem. Before the COVID-19 pandemic, the Global Food Bank Network was “Empowering the World to Defeat Hunger.” Aside from the questionable assertion that expanding charitable food distribution is “empowering,” the claim they are helping to “defeat” world hunger is hyperbolic. While the network reported helping feed 9.6 million people in 34 countries during the 2018/2019 financial year [21], according to the United Nations, the number of people “facing chronic food deprivation” increased by 17 million from 2016 to nearly 821 million in 2017 [22]. While one cannot compare their work in 34 countries to hunger in all 193 nations, it is clear the work is only scratching the surface of the need. Post-pandemic, they changed their motto to “Powering Communities for Zero Hunger,” despite food insecurity doubling worldwide. 

In 2006, the same year the Global Food Bank Network was founded, the World Bank reported that many developed nations had cut social aid programs “in order to discourage use of and prevent long-term dependence on social assistance.” After the 2008 economic collapse, the charitable response to hunger further increased in these countries as further cuts and restrictions were implemented. As the Washington Post reported in 2011, European workers “have been forced to accept salary freezes, decreased hours, postponed retirements and health-care reductions” [23]. Conversely, European corporations often reaped tax credits for their food donations. Even when the world economy boomed again, worker wages in Europe stagnated. 

“As welfare states have retreated,” Riches (2018) asserts, “there can be little doubt that philanthropy, big and small, has privatized emergency food assistance and institutionalized food safety nets at the expense of publicly funded income assistance” (p. 108) [19]. US President George W. Bush promoted “compassionate conservatism” and “faith-based armies of compassion” to reduce poverty while proposing to privatize Social Security. Conservative U.K. Prime Minister David Cameron then proposed similar ideas, a “big society” that coupled government spending reductions with increased volunteerism and a bigger role for charities.” In response, child poverty in Britain soared. 

It is important to note that the term “safety net” in the United States tends to mean a wide variety of means-tested programs that provide specific things (such as food, housing, and healthcare), while in most other industrialized nations the “safety net” provides universal, free healthcare and paid leave, as well as broad-based subsidies for items such as child care and public transportation, alongside significant cash pay-out for unemployed people and/or people experiencing poverty.

Even though the US federal food safety net programs are currently under-utilized and under-funded, the dollar value of food provided by safety net programs—even before their expansion in the pandemic—was more than 15 times that of food provided by all US food bank charities, as calculated by the authors. 

The vital role that food charities currently play in the US should not be overlooked. These organizations are indispensable, particularly given that many of the people they feed are vulnerable immigrants largely excluded from key government aid programs. For now, such charities should continue to exist with as much support as possible, but we cannot delude ourselves into thinking that they could ever solve the problem. 

## 4. Hunger in the US during COVID-19

The COVID-19 global pandemic highlights the effects of public policy on hunger and poverty eradication in the US. Having only just recovered from the hunger crisis caused by the 2008 recession when the COVID-19 outbreak occurred, in March 2020, a new hunger crisis was seen. In 2019, before COVID-19, 10.5 percent of American households were food-insecure, marking the first year that US household food insecurity prevalence was statistically significantly below the pre-recession level of 11 percent in 2007 [24].

While the USDA’s 2020 Household Food Security report [3] showed no significant change in household food insecurity prevalence from the previous year, the number of food-insecure individuals increased by 8.6 percent from 35 to 38 million. The rates represent only a proportion of the year impacted by high unemployment and significant policy changes, as the December survey asks about household food insecurity over the last 12 months, flattening variations in household food insecurity over the course of the year. COVID-19 also affected certain demographic groups differently. The food insecurity rate among children living in food insecure households significantly increased from 14.6 to 16.1 percent. 

### 4.1. Changes to US Federal Food Assistance Policies and Their Impact on Hunger during COVID-19

By examining US data, we can better understand the significant impact policy measures such as increases in SNAP spending, stimulus payments, and the enhanced Child Tax Credit had on preventing greater hunger throughout the pandemic.

The US Census Bureau’s Household Pulse Survey (HPS) that began on 23 April 2020 shows how hunger changed throughout the COVID-19 pandemic [25]. The survey measured how the pandemic affected US households both socially and economically. Phase 1 was collected weekly between 23 April and 21 July 2020, and subsequent phases are collected bi-weekly. The HPS measures food insufficiency over the last week, asking the degree to which the household had enough to eat. Analysis identifies responses of “often not enough to eat;” “sometimes not enough to eat;” and “enough, but not always the kinds of food (I/we) wanted to eat” as food-insufficient, and “enough of the kinds of food (I/we) wanted to eat” as food-sufficient. The USDA’s household food security measures and the HPS’s food sufficiency measures are not directly comparable as the methodology, response rate, and time periods covered are different.

Democratic Party leaders in the US Congress forced a large increase in the federal nutrition assistance programs in 2020 during the pandemic, and spending on the US Department of Agriculture’s domestic food and nutrition assistance programs (the largest ones in the government) in Fiscal Year 2020 reached a historical high of USD 122.1 billion [26].

**Figure 4:**
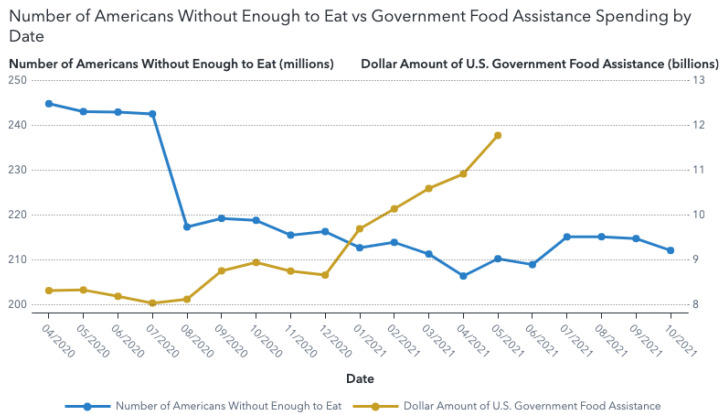
Comparison of the number of food insufficient Household Pulse Survey respondents [25] and the dollar amount of food assistance cash payments (SNAP [28], school lunch, school breakfast, and child and adult care food [29]), April 2020–October 2021. All data used in this graph is publicly available government data.

Figure 4 shows a large decline in food insufficiency, compared with the dollar amount of US spending on the largest domestic food programs between April 2020 and May 2021 including SNAP benefits, school lunch (NSLP), school breakfast (SBP), and child and adult care food (CACFP). These payments increased significantly and consistently after the Biden administration took office in January 2021. While it can be difficult to separate the direct impact of these programs on hunger amidst the numerous changes in the economy resulting from the pandemic, the Census Bureau’s Supplemental Poverty Measure estimates that in 2020, 2.9 million people were lifted out of poverty due to the SNAP program, along with 0.3 million people from the school lunch program [27].

The enhanced Child Tax Credit (CTC), paid to low- and medium-income US families with children, had the most immediate impact on hunger. Compared to before the first payments were received (week 33 of the survey), there was a 5.1 percent decrease in the number of food-insufficient households with children compared to an 8.0 percent increase in the number of food-insufficient households without children (week 36). In week 36, of those who received a CTC payment in the last 4 weeks who had mostly spent their payment (31.7 percent), 68.3 percent said they spent the payment on food [30].

**Figure 5:**
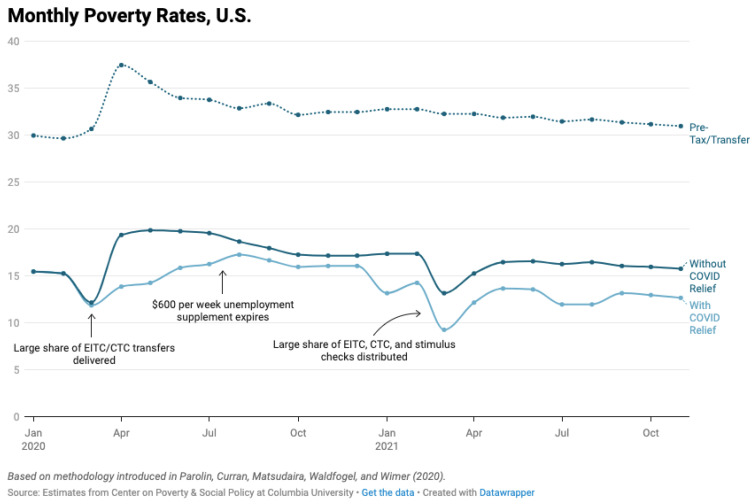
Monthly estimates of the Supplemental Poverty Measure from the Center on Poverty and Social Policy at Columbia University, January 2020–July 2021 [31]. COVID-19 relief efforts such as the stimulus payments prevented higher US poverty rates during the pandemic. Permission for usage of this chart was obtained from Columbia University’s Center on Poverty and Social Policy.

It is estimated that without the additional government COVID-19 stimulus payments, the US poverty rate would have increased by over 25 percent (from 13.9 percent to 19.4 percent) in April 2020 [31]. Furthermore, these estimations illustrate the significant impact the EITC and CTC transfers have in short-term poverty reduction every March (Figure 5). The stimulus cash payments also played a significant role in poverty reduction, lifting an estimated 11.7 million people out of poverty in 2020 [27]. The gap between the estimated poverty rate with and without COVID-19 stimulus payments narrowed to less than 1.5 percentage points between August and December 2020, after the USD 600 unemployment supplement expired in July 2020 [31]. According to the Census Bureau’s Supplemental Poverty Measure, 5.5 million fewer people lived in poverty in 2020 due to unemployment insurance [27].

**Figure 6:**
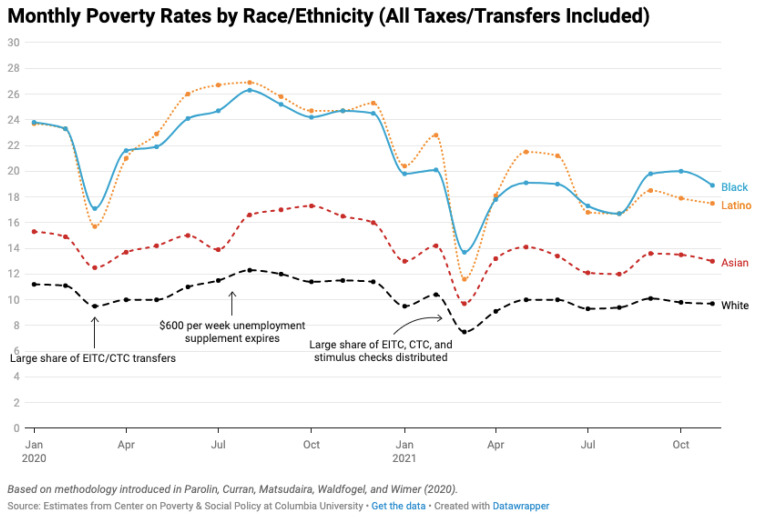
Monthly estimates of the Supplemental Poverty Measure from the Center on Poverty and Social Policy at Columbia University by race/ethnicity, January 2020–July 2021 [31]. Black and Latino poverty levels were more severely impacted by the COVID-19 pandemic than White and Asian populations. Permission for usage of this chart was obtained from Columbia University’s Center on Poverty and Social Policy.

The COVID-19 pandemic impacted demographic groups differently, with more vulnerable populations experiencing larger increases in poverty and hunger. The monthly poverty estimates by race and ethnicity show steeper increases and drops throughout the year for Black and Latino populations compared to more stable trends in Asian and White populations (Figure 6). USDA data also show differences in household food security status from 2019 to 2020 by demographic characteristics. White, non-Hispanic households experienced a statistically significant decrease of 10.1 percent in the rate of food insecurity, while Black, non-Hispanic households experienced a statistically significant increase of 13.6 percent [3]. Statistically significant increases in food insecurity from 2019 to 2020 were seen in households with children over 18 years, married-couple families, and households in the South of the US. Women and men living alone and households in the Midwest all experienced statistically significant decreases in the rate of household food insecurity.

### 4.2. Food Charity Versus the Social Safety Net

Food charities play a vital role in addressing food insecurity; however, their service is dwarfed by the larger impact of bolstering the social safety net. The USDA reported a 53.0 percent increase in the number of households who used food pantries in 2020 [32]. This tracks closely with Feeding America’s figures of a 55 percent increase in the number of people served from before the pandemic [33]. These services meet a critical need, especially considering the barriers many Americans faced when trying to apply for government assistance during the pandemic, such as inaccessible government offices and malfunctioning online application systems. A nationwide survey of Americans who had applied for unemployment and/or SNAP benefits between March and June of 2020 found 24 percent said it was “time consuming and/or difficult to apply” [34].

The economic crisis triggered by the pandemic caused a surge in unemployment and SNAP applications, overwhelming the system and preventing millions of Americans from receiving support. Feeding charities stepped up to fill the unmet need during the crisis; however, these programs faced their own barriers, such as implementing COVID-safe protocols and a lack of staffing/volunteers. In 2020, 11.2 percent of US charity feeding programs were unable to distribute enough food to meet demand, and 22.5 percent turned people away, reduced the amount of food distributed, or limited their hours due to lack of resources [35].

The visual and media impact of the increase in US charity obscured the reality that the role of government dwarfed the role of NGOs in the pandemic. Widespread media coverage using an image of thousands of cars lined up outside a San Antonio, Texas food bank, did not report that at the same time, 294,000 people in Bexar County (where San Antonio is located) received US government-funded SNAP benefits, USD 33.6 million of federal benefits just that month.

SNAP is an entitlement program that is designed to be counter-cyclical, growing when the economy is weak and contracting when the economy is strong. When the federal government took actions to increase the SNAP benefit during the pandemic (due to pushes in 2020 and 2021 by Democratic Party leadership in Congress and 2021 policy changes made by the administration of Democratic President Joe Biden), between February 2020 and June 2021, the number of US residents receiving SNAP increased by 15 percent (from 36.9 to 42.3 million people) and total federal government spending on SNAP increased by 116 percent (from USD 4.5 billion per month to USD 9.7 billion per month) [28].

**Figure 7:**
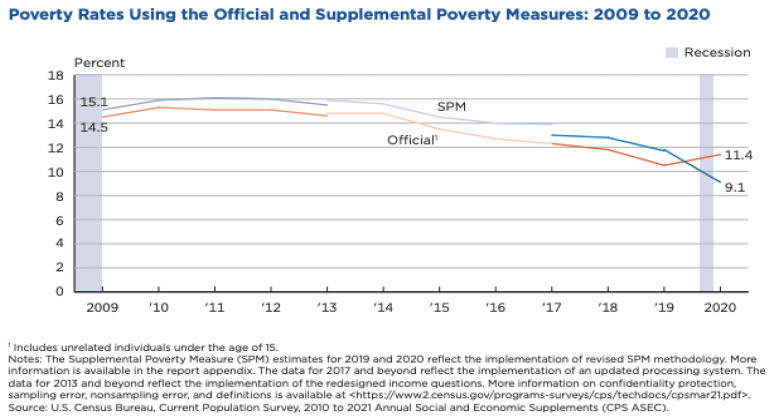
Comparison of the Official and Supplemental Poverty Measures from the US Census Bureau, 2009–2020 [27]. Increased government assistance measures and stimulus payments led to a decrease in the Supplemental Poverty Measure between 2019 and 2020. This figure was created by the US Census Bureau and is in the public domain.

The Census Bureau’s 2020 Supplemental Poverty Measure (SPM) data show the profound effect government intervention can have on poverty reduction. Unlike the Official Poverty Measure (OPM), the SPM takes into account geographic variation, family resources, and expenses, including the increased government assistance measures and stimulus payments. Consequently, 2020 marked the first year the SPM was below the OPM (Figure 7). The OPM increased by 1.0 percentage point in 2020 after experiencing five years of steady decline, while the SPM declined by 2.6 percentage points [27]. SPM estimates for 2021 by the Urban Institute are already predicting poverty will be cut by nearly 50 percent relative to pre-pandemic levels [36]. These figures suggest the degree to which ending poverty is dependent on policy decisions.

## 5. Key Solutions for Reducing Household Food Insecurity and Hunger in the US and Lessons for Other Industrialized Countries

History proves that only effective, focused, domestic political and social movements—powered by the people most impacted by the issue—can achieve big victories. The Poor People’s Movement launched by Dr. Martin Luther King Jr. demonstrates the impact this can have on the hunger movement. When Richard Nixon ran for president in 1968, he essentially denied the existence of US hunger, blaming political opponents for manufacturing a non-existent problem. However, within a year the Poor People’s Movement prompted then-President Nixon to hold the first (and to this day, only) White House Conference on Hunger and to support the creation of the modern federal nutrition assistance safety net. In just a few years after the Poor People’s Campaign was launched, the president and Congress jointly expanded the Food Stamp Program and federal summer meals programs for children from relatively small pilot projects into the National School Breakfast Program and the WIC Program.

Due to government policies aimed at boosting economic opportunity (such as the G.I. Bill, which helped former soldiers pay for college, job training, and first homes) and strong government protections for trade unions, the US also experienced decades of broad-based economic growth following the World War Two. The US grew a vast, prosperous middle class and reduced poverty.

These economic improvements and safety net expansions succeeded in achieving their main goal: ending starvation conditions in America. In 1979, the Field Foundation sent a team of investigators back to many of the same parts of the US with high rates of hunger in the late 1960s. They found dramatic reductions in hunger and malnutrition and concluded “This change does not appear to be due to an overall improvement in living standards or to a decrease in joblessness in these areas… The Food Stamp Program, the nutritional components of Head Start, school lunch and breakfast programs, and…WIC have made the difference.”

In the Reagan era of the 1980s, the US went backwards, outsourcing jobs, crushing unions, limiting wages, and slashing safety net programs. The US and other industrialized nations desperately need new domestic political movements to empower the impoverished masses to take charge of their own futures to end hunger by raising wages and strengthening safety nets.

Most low-income Americans believe the US government could eliminate poverty, homelessness, and hunger [37]. There is broad support for policies to increase the federal minimum wage, guarantee living wage jobs to all adults, ease access to government benefits and banking services, increase spending on SNAP, and eliminate rules under which people lose all their benefits as soon as they work more hours or get a raise.

**Figure 8:**
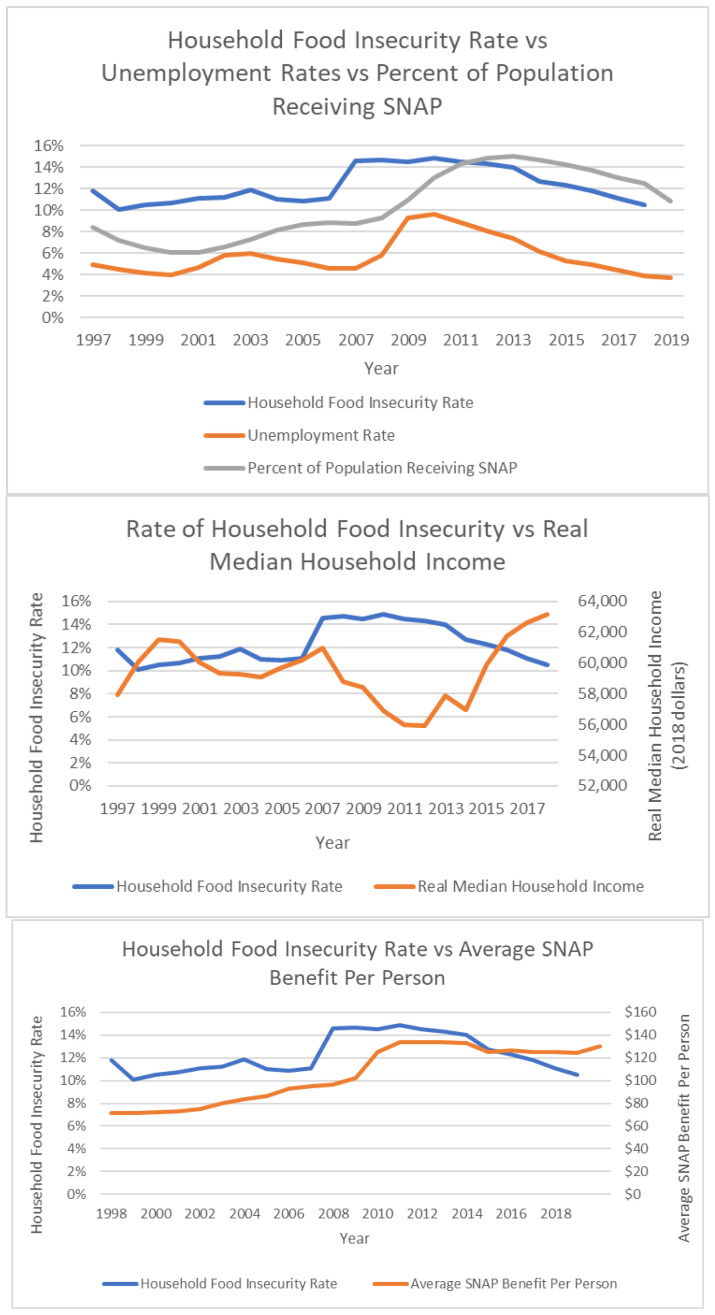
Rate of food household insecurity [3] compared to unemployment rates [38], mean household income [39], and SNAP benefits per person [28], 1998 to 2019. Charts created by Hunger Free America. US employment and wages have the strongest relationship to food insecurity, followed by government nutrition assistance programs.

In the US, employment and wages have the strongest relationship to food insecurity, followed by government nutrition assistance programs (Figure 8).

## 6. Conclusions

It is imperative that the US government continues the anti-hunger legislative momentum built in response to the COVID-19 pandemic. It is not enough to strive to reach pre-pandemic levels of poverty and hunger. History and current events demonstrate that ending hunger in the US is possible with political will. Low-income Americans should not have to wait until the next economic crisis for the government to act.

Hunger Free America has proposed a very detailed federal policy agenda [40]. Our recommendations can very roughly be summarized by these three points vital in ending household food insecurity in the US and industrialized countries.

1. Creating jobs; raising wages; making high-quality healthcare and prescription medicine free; and ensuring that high quality childcare, education, transportation, and broad-band access are affordable.
2. Enacting a comprehensive “Assets Empowerment Agenda” to help low-income people move from owing to owning in order to develop middle-class wealth.
3. When the above two steps are inadequate, ensure a robust government safety net for struggling residents that provides cash, food, and housing assistance.

We again note that many industrialized nations have long taken a number of these steps, and these nations tend to be ones with lower rates of household food insecurity.

History and current events prove that, in the US and elsewhere, a fair economy and a robust safety net do far more to prevent hunger than the public occasionally donating random boxes and cans of food.

## Data Availability

All data generated or analyzed during this study are included in this published article.
